# DNA methylation signatures on vascular differentiation genes are aberrant in vessels of human cerebral arteriovenous malformation nidus

**DOI:** 10.1186/s13148-022-01346-z

**Published:** 2022-10-13

**Authors:** Jaya Mary Thomas, Dhakshmi Sasankan, Mathew Abraham, Sumi Surendran, Chandrasekharan C. Kartha, Arumugam Rajavelu

**Affiliations:** 1 Cardio Vascular Diseases and Diabetes Biology, Rajiv Gandhi Centre for Biotechnology, Poojappura, Thycaud, Thiruvananthapuram, Kerala India 695014; 2 Department of Neurosurgery, Sree Chitra Tirunal Institute for Medical Sciences and Technology, Thiruvananthapuram, Kerala India 695011; 3 Department of Neurology, Amrita Institute of Medical Sciences, Amrita Vishwa Vidyapeetham, Kochi, 682041 Kerala India; 4 Department of Biotechnology, Bhupat and Jyoti Mehta School of Biosciences, Indian Institute of Technology, Madras, Chennai, Tamil Nadu 600036 India

**Keywords:** Epigenetics, DNA methylation, Vascular development, Gene suppression, Arteriovenous malformation, Brain

## Abstract

Arteriovenous malformation (AVM) is a tangle of arteries and veins, rupture of which can result in catastrophic hemorrhage in vulnerable sites such as the brain. Cerebral AVM is associated with a high mortality rate in humans. The causative factor or the stimulus at the artery-venous junction and the molecular basis of the development and progression of cerebral AVM remain unknown. While it is known that aberrant hemodynamic forces in the artery-vein junction contribute to the development of AVMs, the mechanistic pathways are unclear. Given that various environmental stimuli modulate epigenetic modifications on the chromatin of cells, we speculated that misregulated DNA methylome could lead to cerebral AVM development. To identify the aberrant epigenetic signatures, we used AVM nidus tissues and analyzed the global DNA methylome using the Infinium DNA methylome array. We observed significant alterations of DNA methylation in the genes associated with the vascular developmental pathway. Further, we validated the DNA hypermethylation by DNA bisulfite sequencing analysis of selected genes from human cerebral AVM nidus. Taken together, we provide the first experimental evidence for aberrant epigenetic signatures on the genes of vascular development pathway, in human cerebral AVM nidus.

**Supplementary Information:**

The online version contains supplementary material available at 10.1186/s13148-022-01346-z.

## Introduction

Vascular malformations are deformities in blood vessels and can occur in any part of the different types of a blood vessel such as artery, vein, or capillary. They are classified as high flow or low flow types. Arteriovenous malformations (AVMs) are high flow types characterized by a low resistance shunt pathway [32]. In AVMs, the normal capillary structures in between arteries and veins are absent and instead, a tangle of aberrant blood vessels called nidus is present [24]. Earlier studies suggested that AVMs are congenital defects. Subsequent studies clearly indicate de novo development of AVM [8, 24]. AVMs can occur in any part of the human body. AVMs in the brain are at high risk for increased morbidity and mortality because of serious hemorrhage and its associated complications [14]. The surgical removal of the AVMs nidus is the definitive treatment to cure the disease. Embolization through blood vessels and radiation therapy can be used independently or as adjuncts with surgery to manage select subgroups groups of cerebral AVMs. High-grade AVMs are still considered challenging lesions from a therapeutic point of view. The molecular mechanisms for pathogenesis and causative factors for the development of cerebral AVMs remain elusive. Reports have indicated that altered hemodynamic forces at the artery–vein junction may trigger the development of AVMs [27]. Understanding the disease pathways that lead to structural changes in AVM nidus could pave way for identifying strategies to prevent the recurrence of AVM after their surgical removal.

Epigenetic mechanisms have a significant role in regulating gene expression without alterations in the DNA sequence. Three major epigenetic players such as DNA methylation, histone modifications, and lncRNAs are the key regulators that control cellular growth and differentiation [4]. Among them, DNA methylation primarily occurs in the gene promoters at the 5th base of cytosine in CpG dinucleotides and mediates gene suppression [22]. DNA methylation involves covalent modification by transfer of methyl group from S-adenosyl-L-methionine (SAM) cofactor to the 5th carbon of cytosine base on the DNA by three major DNA methyltransferases such as DNMT1, DNMT3A, and DNMT3B [15, 19, 23]. The DNA methylation pattern varies among tissues, and aid to maintain the unique DNA methylation pattern during the development of organisms. Fine-tuning of DNA methylation is highly important for the normal development of an organism [10]. Aberrant DNA methylation patterns on gene promoters are commonly observed in many chronic diseases, metabolic diseases, and vascular diseases [12], suggesting that regulation of DNA methylation signatures on gene promoters is essential for normal cellular development.

Vascular diseases such as vascular anomalies, arteriovenous malformations, and varicose can vary from a simple deformation of vascular structures to complex structures and life-threatening diseases. The molecular basis of the development of these vascular diseases is poorly understood. Recent studies have revealed the role of non-genetic components in the pathogenesis of the vascular disease. Identical twins who share a common genetic background can have varied vascular disease pathogenesis [29]. This observation indicates the role of epigenetic players in the development of vascular disorders, as the epigenetic changes are regulated by environmental stimuli.

The vascular system is subject to varying degrees of abnormal hemodynamic flow that may trigger alterations in the epigenome and eventually to the development of vascular abnormalities [35]. Epigenetic modifications, especially DNA methylation are now recognized to play an important role in normal as well as pathologic vascular development [21]. The human global methylation pattern has revealed the involvement of hypermethylation of DNA in inflammatory conditions [7].

Aberrant expression of genes involved in vasculogenesis and inflammation has been observed in the nidus structure of cerebral AVMs [36]. The pathways that lead to the altered gene expression are unclear. Altered cerebral hemodynamic flow has been speculated to contribute to the pathogenesis of high flow [11, 35]. We proposed that alterations in hemodynamic forces could result in epigenetic changes in vascular cells [35]. Whether aberrant epigenetic modifications are present in AVM nidus structures has not been explored. Wang and colleagues recently reported that Wilms’ tumor 1-associating protein (WRAP), one among the epi-transcriptome proteins is down-regulated in brain AVM lesions [39], epigenetic modifications have not been identified.

In our present study of cerebral AVM nidus tissues, we observed the presence of aberrant DNA methylation signatures on the genes associated with vasculogenesis. We have substantiated our findings by promoter DNA methylation analysis of cerebral AVM nidus. To our knowledge, the presence of aberrant DNA methylation in the cerebral AVM nidus is reported for the first time. Aberrant DNA modifications could deregulate the vascular developmental pathway genes and thus contribute to the development of cerebral AVMs in humans.

## Results

### Clinical and pathological examination of human cerebral AVM nidus

**Fig. 1:**
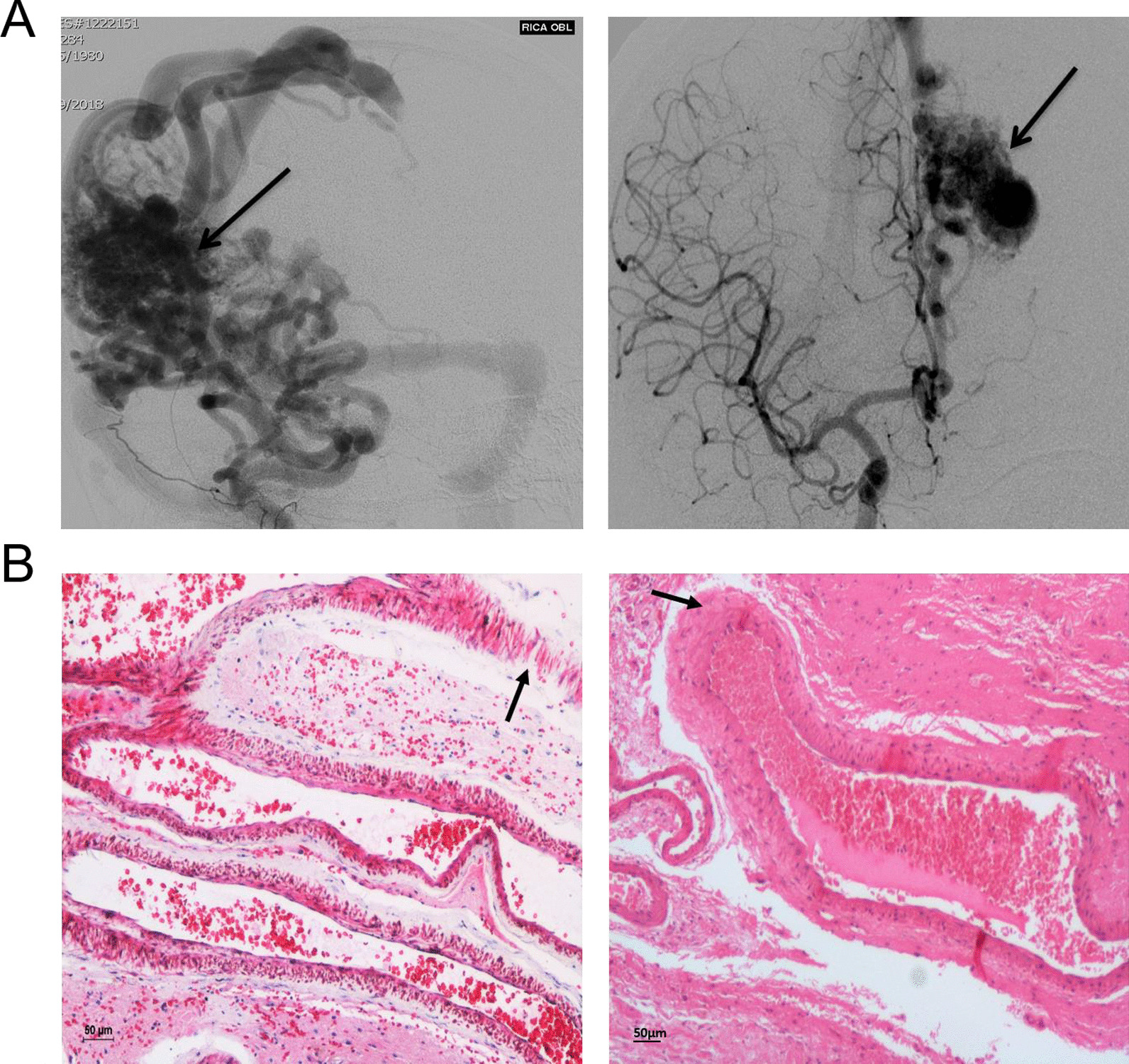
**a** Representative images of cerebral angiogram reveals that the abnormal AVM nidus at the cerebral region. The arrow represents the nidus portion of AVM. **b** Large abnormal blood vessels were seen in Hematoxylin and Eosin staining of brain AVM nidus. A distorted vein like vessel with partial denudation of endothelial cell layer (left) and large vein with proliferation in tunica media layer was observed using 10X magnification (right)

Patients with typical symptoms of cerebral AVM, such as severe headache, numbness, vision loss, and difficulty in speaking were subjected to digital subtraction angiography (DSA) and thus the diagnosis of AVM was confirmed. We used AVM nidus samples from two patients for global DNA methylome analysis. One sample was from an AVM in the right parietal lobe and of high flow Spetzler Martin Grade III. AVM was nearly 5 cm in size and compact. The second sample was from an AVM located in the left medial frontal lobe and of Spetzler Martin grade I, 2 cm in size (Fig. 1a). The samples were collected carefully during surgery and transported to the laboratory. The pathological nature of AVM tissues was confirmed by Hematoxylin–Eosin staining and microscopy, which revealed the tortuous vessels characteristic of brain arteriovenous nidus (Fig. 1b). There were both ruptured and unruptured vascular structures in the AVM nidus samples and it is not an acute rupture and some parts of the vessels ruptured prior to the surgery (Fig. 1b). The cerebral AVM nidus is comprised of thin-walled and thick-walled vessels. Enlarged veins with severely thickened tunica media were present. Intimal hyperplasia caused occlusion in vessels at several sites. Infiltration of inflammatory cells in the brain parenchyma and vessels was evident, especially in the ruptured nidus structures (Fig. 1b).

### Analysis of global differential DNA methylation in cerebral AVM nidus

**Fig. 2:**
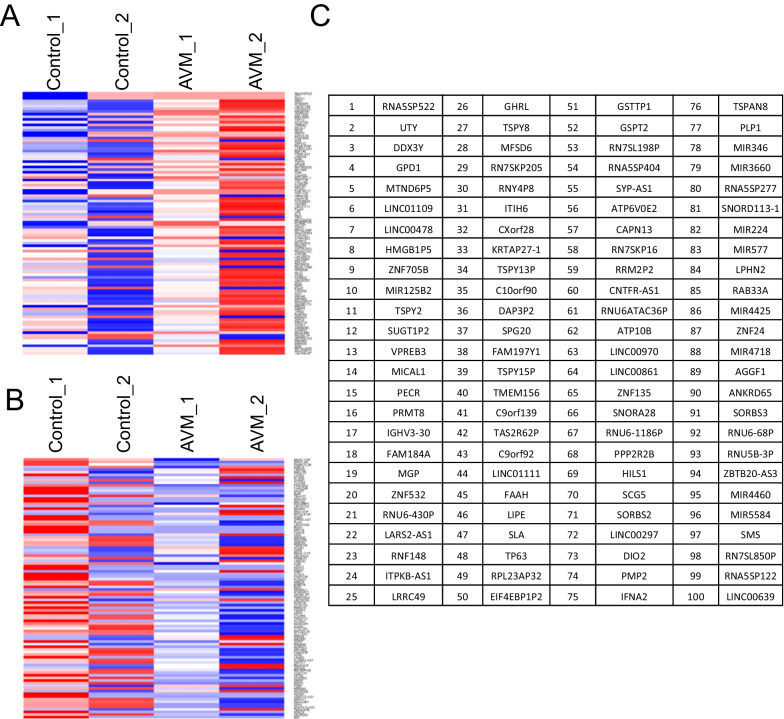
Infinium bead ChIP array for DNA methylation analysis. **a** The heatmap represent the the hypermethylated CpGs located in the promoter regions of genes. We have two control tissues and two AVM tissues and top 100 hypermethylated genes were used to generate the heatmap. The red color indicates the hypermethylation, and blue color indicates the DNA hypomethylation. There were significant number of CpGs were shown hypermethylation. **b** The heatmap represents the hypomethylated CpGs of the cerebral AVM nidus. There were not much significant DNA hypomethylation was observed among the duplicate’s samples of AVM. **c** The list of hypermethylated CpGs of AVM nidus and its nearest genes, the list is extracted from (**a**)

To investigate the aberrant DNA methylation changes in cerebral AVM nidus, total DNA was isolated from cerebral AVM nidus and control vascular tissues. The DNA was processed for bisulfite conversion and subjected to an Infinium methylome array. The differentially methylated CpG located in the promoter regions were selected and the nearest genes were identified. We have selected the top 100 differentially methylated genes. The beta values calculated from methylated and unmethylated cytosine fluorophore from the array were used to identify the hyper and hypomethylated genes and taken as input for generating the heatmaps (Fig. 2) (Additional file 2). The beta value for each CpG site with more than 0.5-fold than the control sample is considered hypermethylation, whereas less than 0.5-fold change than the control sample is considered hypomethylation. We found a significant number of genes that are directly associated with vascular development are hypermethylated in AVM nidus than control samples (Fig. 2). We did not however observe a significant number of hypomethylated genes in AVM nidus (Fig. 2). The global DNA methylome screening identified the presence of aberrant DNA methylation signatures in the cerebral AVM nidus.

### Promoter methylation analysis confirms the aberrant DNA methylation signatures on the vascular pathway-specific genes

**Fig. 3:**
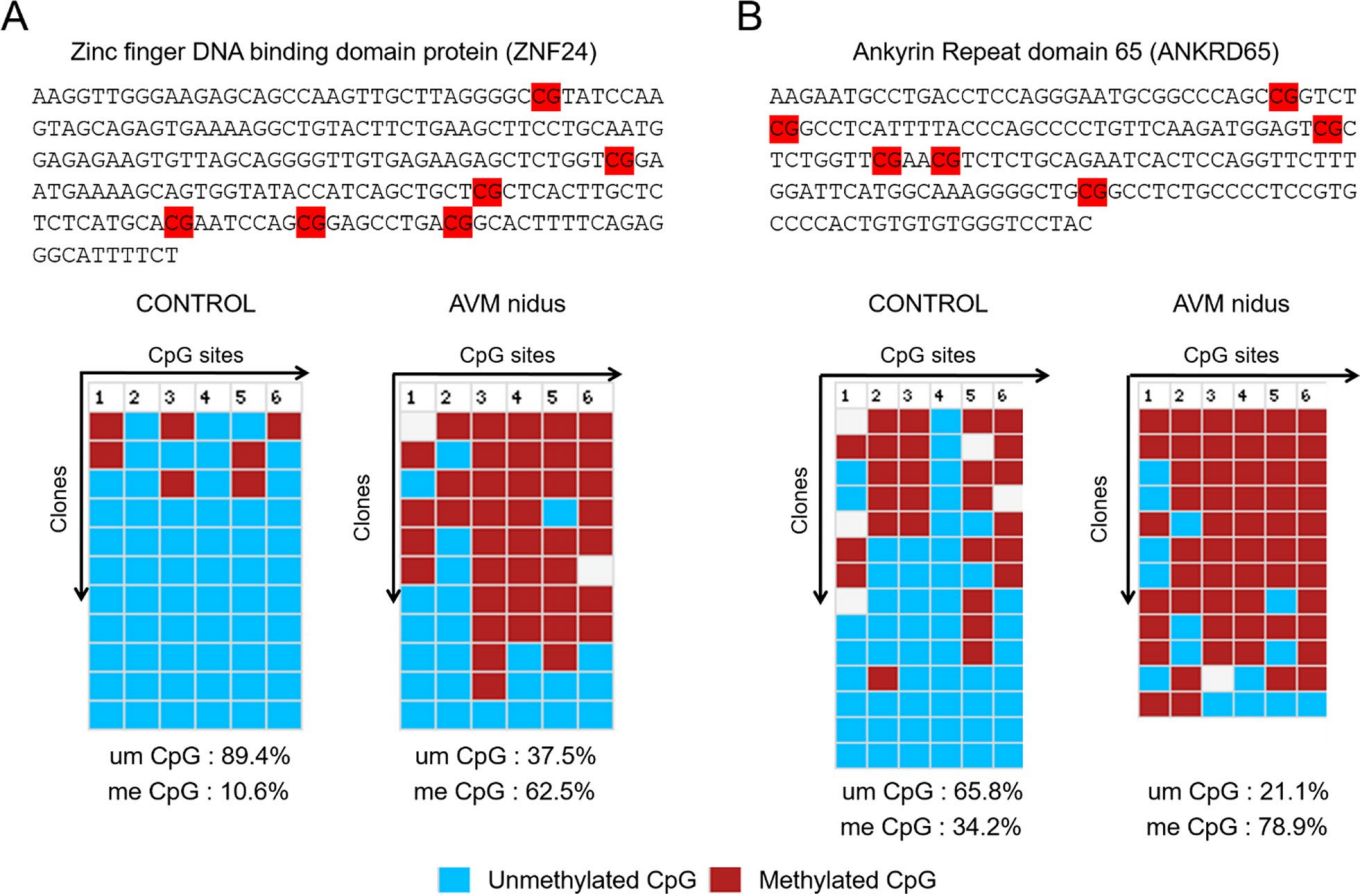
Promoter DNA methylation analysis by bisulfite conversion and sequencing. **a** The selected promoter region of ZNF24 gene for methylation analysis, and the CpGs are marked in red color. The bottom image represents the output from the BISMA analysis (Rhode et al. 2009) of clones prepared from control and AVM nidus samples. The column number represents the CpG and the clones represents the amplicons. **b** The CpGs in ANKSRD65 gene promoter are marked in red color and the bottom results is the output from BISMA analysis of the clones. The clones amplified from cerebral AVM has the significantly higher number of methylated CpG than control tissue. The blue color box represent the unmethylated CpG and red color box represent the methylated CpG. Percentage of methylated CpGs are provide at the bottom of the image

**Fig. 4:**
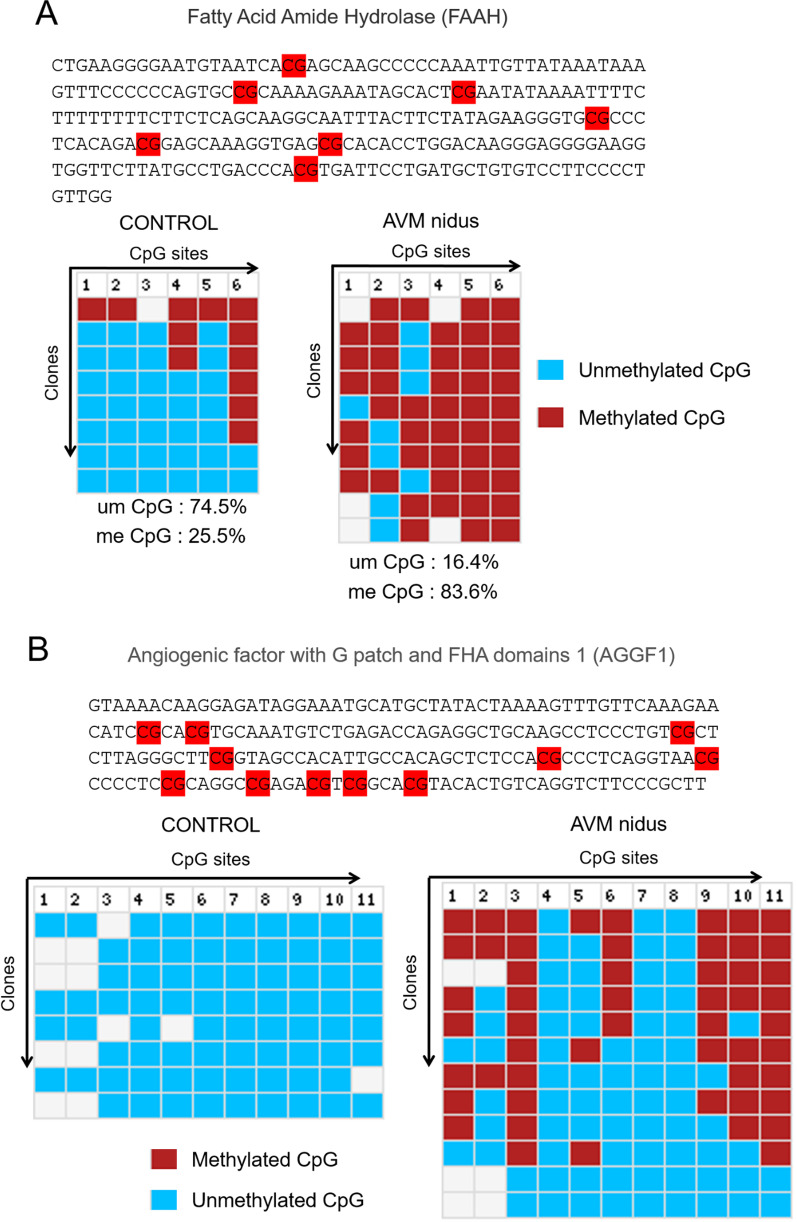
Promoter DNA methylation analysis by bisulfite conversion and sequencing. **a** The selected promoter region of FAAH gene, and the CpGs are marked in red color. The bottom image is the result from the BISMA analysis of clones amplified from control and AVM nidus samples after bilsulfite conversion. The column number represents the CpG and the clones represent the amplicons. **b** The CpGs in AGGF1 gene promoter are marked in red color and the results of the clones amplified from control and AVM tissues from BISMA analysis. The blue color box represent the unmethylated CpG and red color box represent the methylated CpG. Percentage of methylated CpGs are provide at the bottom of the image

The Infinium methylome array results provide the methylation status of a single CpG of the gene promoter; however, it will not provide the methylation status at the sequence level. To substantiate the DNA hypermethylation, we used DNA isolated from AVM nidus and control tissue (3rd sample) and performed the promoter DNA methylation analysis for the CpG islands of selected genes. We performed bisulfite conversion and sequencing of the selected gene promoters, which are known to involve in vascular development [16, 17, 25, 31, 38]. We selected four genes such as ZNF24 (also known (Kox17), AGGF1 (Angiogenic factor with G patch and FHA domains 1), ANKRD65 (Ankyrin repeat domain 65), and FAAH (Fatty acid amid hydrolase) for further validation of DNA hypermethylation on the promoter of these genes [16, 17, 25, 31, 38]. For each gene, we analyzed more than 10 clones. The DNA bisulfite sequencing analysis has found that significant levels of DNA hypermethylation on the CpGs of the ZNF24 and ANKRD65 genes promoter of AVM nidus than in control tissues, importantly more than 50% of the CpGs are methylated in AVM nidus (Fig. 3a, b). Similarly, we also found DNA hypermethylation on the CpGs of FAAH and AGGF1 gene promoters. We observed more than 60% of CpGs are methylated in the FAAH gene promoter and nearly 100% DNA methylation on the CpG of the AGGF1 gene promoter in AVM nidus (Fig. 4a, b). It is evident that the presence of DNA hypermethylation on these subsets of genes in cerebral AVM nidus, which could contribute to the development of cerebral AVM in humans. The aberrant epigenetic modification on the gene promoter could be the triggering factor in the development of cerebral AVM.

## Discussion

Cerebral AVMs are considered to develop because of high and aberrant hemodynamic flow at the junction of arteries and veins, resulting in enlarged vascular structures. These AVMs rupture and bleed with a high risk of death in the patient [9, 28, 33]. The causative factors for the development of cerebral AVMs remain unknown. Alterations in the hemodynamic flow at the juncture of the feeding artery and draining vein may induce epigenetic changes in endothelial cell genes to deregulate vascular development [35]. Chen et al. identified DNA methylation changes in the cyclin-dependent kinase inhibitor 2A (CDKN2A) gene in an analysis of blood samples of patients with cerebral AVM [6]. CDKN2A gene codes for a well-known tumor suppressor protein and does not link aberrant hemodynamic changes with AVM development. In our study of tissues of cerebral AVMs, we found the presence of aberrant DNA methylation signatures on the promoters of subsets of genes that are associated with the vascular development pathway (Fig. 3). The promoter DNA methylation analysis confirmed that the observed DNA hypermethylation of these genes is quite significant (Figs. 3, 4). The external stimuli are known to drive the epigenetic changes, viz. DNA methylation and histone methylation on the chromatin of cells [1–3]. The altered epigenetic changes can reprogram gene expression in endothelial cells in response to environmental stimuli. Aberrant hemodynamic flow at the junction of the artery and vein could potentially alter epigenetic factors in vascular endothelial cells.

We report the presence of DNA hypermethylation in the promoter of vascular-specific genes namely ZNF24, FAAH, and AGGF1, in cerebral AVM nidus. vascular endothelial growth factor (VEGF) is the major mediator of angiogenesis in both physiological and pathological conditions [13]. There is increased expression of VEGF(A-D) and their receptors, Flt-1, Flk-1, and Flt-4 in the vascular endothelial cells of AVM and in astrocytes in the neighborhood of AVM [20]. ZNF24 is a well-known transcriptional repressor of the VEGF gene. Overexpression of the ZNF24 gene suppresses angiogenesis in tumors [16]. We observed that more than 50% of the CpGs of the ZNF24 gene promoter are hypermethylated in AVM tissue which suggests downregulation of ZNF24. Suppression of the ZNF24 gene in AVM nidus could activate transcription of VEGF and may trigger AVM development.

FAAH is involved in hypoxic pulmonary vasoconstriction through the production of various vasoconstrictive eicosanoids [40]. Inactivation of FAAH could attenuate both in vitro and in vivo angiogenesis [25]. We found that more than 60% of CpGs in the gene promoter are hypermethylated in cerebral AVMs. This can lead to decreased expression of FAAH and thus contribute to AVM pathogenesis.

AGGF1 is a potent regulator of vascular development and angiogenesis. AGGF1 modulates all stages of vascular development such as endothelial cell proliferation, migration, and endothelial cell fate determination to the vein. Similar to VEGF, AGGF1 also regulates both physiological and pathological angiogenesis [37]. Knockdown of the AGGF1 gene results in severe embryonic lethality, impairs blood vessel growth in the yolk sac, and suppress vein formation [4]. Overexpression of AGGF1 promotes venous identity by inducing the expression of vein marker flt4 [4]. Cerebral AVM vessels overexpress vein fate determinant COUP TF-II [30]. We observed 100% DNA hypermethylation on the AGGF1 gene promoter in AVM tissues which suggest a decreased expression of the AGGF1 gene that could lead to suppression of vein formation.

It is essential to consider that conclusion was drawn from the results obtained using 2 AVM and control samples in an Infinium array and further promoter DNA methylation for 4 target genes was validated using 3rd AVM and control samples (Figs. 3, 4). Although the observed results were quite consistent among all 3 AVM samples, further validation using additional sets of AVMs tissues will provide a larger conclusion. We have used AVM nidus structure to prepare the genomic DNA for bisulfite conversion, and pathological changes in these structures were confirmed (Fig. 1). Since the AVM and control tissues are from different individuals, it is possible these aberrant nidus vessels may carry a little healthy vascular structure which may also bring the heterogeneity among the samples. An important question that remains unanswered is, at what stage of development, vascular structures are prone to epigenetic changes caused by aberrant blood flow. Earlier studies have indicated that cerebral AVM nidus has elevated expressions of capillary, artery, and vein-specific marker proteins, which suggest the cerebral AVMs are deficient in terminal differentiation of vascular structures [35, 36]. As aberrant DNA methylation contributes to the development of cerebral AVMs, reversal of DNA methylation using DNMT-specific inhibitors (DNMTi) could be useful in the prevention of recurrence of the AVMs.

## Materials and methods

### Ethics statement

The cerebral AVM nidus and control vascular tissues were collected as per prescribed guidelines and the study was approved by the Institutional Ethical Committees of both Sree Chitra Tirunal Institute for Medical Sciences & Technology (SCTIMST) where patients were treated and Rajiv Gandhi Centre for Biotechnology (RGCB), where the studies were done.

### Cerebral angiogram and surgical removal of AVM nidus

The gold standard investigation to elucidate the exact extent and nature of a cerebral AVM is a digital subtraction angiogram (DSA). The most popular and therapeutically valuable classification of AVMs has been the Spetzler Martin grading based on the AVM dimensions, venous drainage and eloquence of the location [34]. An AVM has arterial feeders, a nidus and draining veins. Total elimination of the AVM without producing unacceptable sequalae is the therapeutic goal. Surgery involves the excision of the AVM nidus after cutting off the arterial feeders and ensuring that the draining vein is no longer an active shunt. Samples were collected from the excised AVM nidus of patients who underwent surgical resection in the institute for this condition.

### AVM fixation, sectioning, H and E staining

AVM nidus and control brain specimens were fixed in 10% neutral buffered formalin and embedded in paraffin wax. The paraffin-embedded tissue blocks were sectioned and 4-μm-thick sections were deparaffinized by xylene. Tissue sections were further rehydrated using graded concentrations of ethanol in water. Sections were stained with hematoxylin (Sigma Cat # 105175) and eosin (Sigma Cat # 230251) and observed under brightfield microscope (Nikon, Japan) for histological analysis.

### Cerebral AVM nidus collection and genomic DNA isolation

Control tissues consisted of brain tissue from epileptic patients who underwent corrective surgery. The total genomic DNA was isolated using AllPrep DNA/RNA Mini kit (Qiagen Cat No. 80204) as per the manufacturer’s instructions. The quality and concentration of genomic DNA were measured using agarose gel electrophoresis and Nanodrop, 2000 (Additional file 1: Fig. 1).

### Global DNA methylome analysis using Infinium array

We performed the global DNA methylome analysis with 2 control and 2 AVM nidus tissues. The Infinium methylation EPIC bead chip array with 850,000 genome wide methylation sites was used to study the genome wide DNA methylation analysis for the genomic DNA isolated from the AVM nidus and control tissues. This array provides unparalleled coverage of CpG islands, genes, and enhancers at single base resolution. For bisulfite conversion, the genomic DNA was fragmented and subjected for bisulfite conversion. The DNA samples were prepared using Infinium HD methylation assay kit. The fragmented and bisulfite-converted DNA molecules anneal to locus specific DNA oligomers linked to individual bead types. The single base extension of the probes was carried out using dinitrophenyl or biotin labeled dNTPs (A and T nucleotides are dinitrophenyl labeled; C and G nucleotides are biotin labeled). During bisulfite conversion the unmethylated cytosines bases are deaminated to uracil, while methylated cytosines remains as cytosine. Further, the array was fluorescently stained, scanned using Illumina iScan, and the intensities of the methylated and unmethylated bead types were measured.

### Methylation array data analysis

The intensities of the methylated and unmethylated bead types were measured, and the Illumina Genome Studio software was used to analyze the raw data. The DNA methylation values were recorded as beta values and it is variable between 0 and 1, which represent the ratio of the intensity of the methylated bead type to the combined locus intensity. The proportion of CpG loci with significant differential methylation i.e., with beta values was compared between control samples and AVM nidus samples. The loci with significant differential DNA methylation were represented as a percentage of methylation in control versus AVM nidus samples. The genes with nearest to significant DNA methylation changes of loci were identified. The differential methylation on the single CpG site level was computed based on FDR adjusted *p* value < 0.05 to assess whether the methylation values in the two groups originate from distinct distributions. For differential methylation for the different regions (Tiling, genes, promoters and CpG islands) also we computed based on FDR adjusted *p* value < 0.05. The heatmaps were plotted using p values. The top 100 genes for the promoter region were selected using standard deviation of the two samples. Based on the beta values hyper and hypomethylated genes were selected and taken as input to generate the heatmaps. The hypermethylated CpGs should be more than 0.5 in the test than control, whereas for hypomethylated CpGs the test should be less than 0.5 compared to control.

### Promoter DNA methylation analysis by sodium bisulfite conversion

We have used 3rd set of AVM nidus and control tissues to validate promoter DNA hypermethylation on the selected genes. The genomic DNA isolated from control and AVM nidus was subjected to sodium bisulfite conversion as described earlier (Zhang et al. 2009). The genomic DNA was digested with restriction endonuclease enzyme and mixed with 187 µl of Solution I (0.95 g NaHSO_3_ in 1.25 ml water and 325 µl 2 M NaOH). Further, 73 µl of freshly prepared solution II (98.6 mg of 6-hydroxy-2,5,7,8-tetramethylchroman-2-carboxylic acid (Sigma cat # 238813) is dissolved in 2 mL of Dioxane) was added and thoroughly mixed by pipette. The DNA samples were subjected to bisulfite conversion using following reaction conditions in thermocycler: 15 min at 99 °C, 30 min at 50 °C, 5 min at 99 °C, 90 min at 50 °C, 5 min at 99 °C, and 1.5 h at 50 °C. The converted DNA samples were mixed with 150 μL of sterile distilled water and purified using Ultracel YM-50 columns. The bisulfite-converted DNA samples were eluted using 1X TE buffer, stored at − 20 °C and the concentration of DNA samples were measured using Nanodrop 2000.

### Amplification and sequencing of selected gene promotes from bisulfite-converted DNA

To amplify the selected region at the promoter of genes, we used sodium bilsulfite converted DNA specific primers (Additional file 1: Table 1). Following PCR conditions were followed to amplify the promoters: 2 μL of the bisulfite-converted DNA was used as template for PCR in a 25 μL reaction mixture (1 × PCR buffer, 1.5 mM MgCl2, 0.2 mM of each dNTP, 0.4 μM of each primer and 2.5U of HotStarTaq polymerase). The PCR was performed with the following program: 15 min at 95 °C, 5 × (30 s at 94 °C, 30 s at 55 °C, 90 s at 72 °C), 5 × (30 s at 94 °C, 30 s at 50 °C, 90 sat 72 °C), 35 × (30 s at 94 °C, 30 s at 45 °C, 90 s at 72 °C), final extension 10 min at 72 °C. The quality and successful amplification of specific fragments were analyzed on 8% 8% PAGE gel and stained with gel red to visualize the PCR product under UV-light. The PCR products further purified were purified using MN PCR DNA clean-up kit. The DNA sequencing for each amplicon was carried out using the BigDye® Terminator v3.1 cycle sequencing kit with specific primers (Additional file 1: Table 1). The sequencing PCR reaction was mixed with 2.5 µL of 125 mM EDTA and 10 µL of 3 M sodium acetate (pH: 4.8) and 100 µL of water and 260 µL of 100% ethanol and incubated for 20 min and centrifuged for 25 min at 11,000 rpm at room temperature. The precipitated DNA samples were washed using 70% ethanol wash and air dried at the dark condition and reconstituted and sequenced using 3730 DNA Analyzer (Thermo Fisher Scientific).

### Bisulfite sequencing DNA sequencing analysis using BISMA

The raw DNA sequencing file was processed by using BioEdit Sequence Alignment Editor (Ibis Biosciences, Carlsbad, CA). The DNA sequences were further filtered for sequence identity, conversion rate and clonal sequences with default parameters by using Bisulfite Sequencing DNA Methylation Analysis (BISMA) Software (Rohde et al. 2010), and Bisulfite Sequencing Data Presentation and Compilation (BDPC) web interface (Rohde et al. 2008). The BISMA program calculates the percentage of methylation with its default setting parameter. Minimum of 10 clones for each sample were analyzed to obtain the statistical significance between control and AVM samples.

## Supplementary Information

**Additional file 1. Supplementary figure 1:** The agarose gel electrophoresis of the genomic DNA isolated from AVM and control tissues confirms the intactness of DNA isolated from all the samples which were used for Infinium methylome array. The quality and concentration of these DNA samples are provided in the table. **Supplementary table 1**: List of bisulfite primers.

**Additional file 2. Supplementary Table 2:** Differentially methylated promoter level.

## Data Availability

All the data generated in this study are included in the manuscript and Additional file 1 and 2.
