# Supplementary material for: DNA methylation signatures on vascular differentiation genes are aberrant in vessels of human cerebral arteriovenous malformation nidus
Source: Clin Epigenetics. 2022 Oct 13;14:127. doi: 10.1186/s13148-022-01346-z (PMC9563124; doi:10.1186/s13148-022-01346-z)
Supplement: Supplementary file 1 — Additional file 1. Supplementary figure 1: The agarose gel electrophoresis of the genomic DNA isolated from AVM and control tissues confirms the intactness of DNA isolated from all the samples which were used for Infinium methylome array. The quality and concentration of these DNA samples are provided in the table. Supplementary table 1: List of bisulfite primers. [file 13148_2022_1346_MOESM1_ESM.docx]

**Supplementary information for**

**DNA methylation signatures on vascular differentiation genes are aberrant in vessels of human cerebral arteriovenous malformation nidus**

**Jaya Mary Thomas^1#^, Dhakshmi Sasankan^4#^, Mathew Abraham^2^, Sumi Surendran^1^, Chandrasekharan C Kartha^3^*, Arumugam Rajavelu^4^***

1. Cardio Vascular Diseases and Diabetes Biology, Rajiv Gandhi Centre for Biotechnology, Poojappura, Thycaud, Thiruvananthapuram, Kerala, India, 695014.
2. Department of Neurosurgery, Sree Chitra Tirunal Institute for Medical Sciences and Technology, Thiruvananthapuram, Kerala, India, 695011.
3. Department of Neurology, Amrita Institute of Medical Sciences, Amrita Vishwa Vidyapeetham, Kochi, 682041, Kerala, India.
4. Department of Biotechnology, Bhupat & Jyoti Mehta School of Biosciences, Indian Institute of Technology, Madras, Chennai, Tamil Nadu, 600 036, India.

*Correspondence to be addressed: [cckartha@gmail.com](mailto:cckartha@gmail.com) & [arumugam.rajavelu@iitm.ac.in](mailto:arumugam.rajavelu@iitm.ac.in)

**# Shared first authors**

**Running title:** Aberrant DNA methylation in human cerebral AVMs

**Supplementary figure 1:** The agarose gel electrophoresis of the genomic DNA isolated from AVM and control tissues confirms the intactness of DNA isolated from all the samples which were used for Infinium methylome array. The quality and concentration of these DNA samples are provided in the table.

**Supplementary table 1: List of bisulfite primers**

| **S. No** | **Name of gene** | **Primer seq (5’ – 3’)** | **Amplicon size** |
| --- | --- | --- | --- |
| 1 | ZNF24_FP | AAGGTTGGGAAGAGTAGTTAAGTTGT | 225 bp |
| 2 | ZNF24_RP | AAAAAAATACCCTCTAAAAAATACC |  |
| 3 | ANKRD65_FP | AAGAATGTTTGATTTTTAGGGAATG | 182 bp |
| 4 | ANKRD65_RP | ATAAAACCCACACACACAATAAAACAC |  |
| 5 | FAAH_FP | TTGAAGGGGAATGTAATTAT | 250 bp |
| 6 | FAAH_RP | CCAACAAAAAAAAAAACACAACATC |  |
| 7 | AGGF_FP | GTAAAATAAGGAGATAGGAAA | 207 bp |
| 8 | AGGF_RP | AAACRAAAAAACCTAACAATATAC |  |
